# Mechanisms of Epidermal Growth Factor Effect on Animal Intestinal Phosphate Absorption: A Review

**DOI:** 10.3389/fvets.2021.670140

**Published:** 2021-06-14

**Authors:** Xiaopeng Tang, Xuguang Liu, Hu Liu

**Affiliations:** ^1^State Engineering Technology Institute for Karst Desertfication Control, School of Karst Science, Guizhou Normal University, Guiyang, China; ^2^State Key Laboratory of Grassland Agro-Ecosystems, International Centre for Tibetan Plateau Ecosystem Management, Engineering Research Center of Arid Agriculture and Ecological Remediation of Ministry of Education, School of Life Sciences, Lanzhou University, Lanzhou, China

**Keywords:** epidermal growth factor, NaPi-IIb, phosphate uptake, phosphorus, small intestine

## Abstract

Phosphorus is one of the essential mineral elements of animals that plays an important role in animal growth and development, bone formation, energy metabolism, nucleic acid synthesis, cell signal transduction, and blood acid–base balance. It has been established that the Type IIb sodium-dependent phosphate cotransporters (NaPi-IIb) protein is the major sodium-dependent phosphate (Pi) transporter, which plays an important role in Pi uptake across the apical membrane of epithelial cells in the small intestine. Previous studies have demonstrated that epidermal growth factor (EGF) is involved in regulating intestinal Pi absorption. Here we summarize the effects of EGF on active Pi transport of NaPi-IIb under different conditions. Under normal conditions, EGF inhibits the active transport of Pi by inhibiting the expression of NaPi-IIb, while, under intestinal injury condition, EGF promotes the active absorption of Pi through upregulating the expression of NaPi-IIb. This review provides a reference for information about EGF-regulatory functions in Pi absorption in the animal intestine.

## Introduction

Phosphorus is one of the most abundant elements in mammals involved in a variety of physiologic processes in the form of inorganic phosphates (Pi), including cellular signaling, energy metabolism, and nucleotide biosynthesis, and is an important component of cell membranes and bones (1–4). As an important site for Pi absorption, the small intestine plays a crucial role in Pi homeostasis, which accounts for more than 70% of the Pi absorption (5). It is well-known that intestinal Pi absorption by the paracellular route, a non-hormonally-dependent process that occurs mainly through the tight junctions by passive diffusion and the transcellular pathway, occurs through sodium-dependent phosphate co-transporters present in the cell membrane (6–8). Previous studies have demonstrated that active absorption of Pi is mediated by the sodium-dependent transport, SLC34 families, and type II sodium dependent phosphate cotransporters (NaPi-II) (9, 10). NaPi-IIb-mediated Pi transport across the epithelial apical membrane is the main form of Pi uptake in the small intestine (5, 11). NaPi-IIb was first found in mice by Hilfiker in 1998 and confirmed that NaPi-IIb was mainly expressed in the brush border membranes (BBMs) of intestinal epithelial cells (12). Subsequently, researchers have cloned NaPi-IIb in human (13), rat (14), goat (15), chicken (16), and pig (5) and conducted comprehensive studies on the factors affecting its expression in the small intestine.

Epidermal growth factor (EGF), a small mitogenic polypeptide comprising 53 amino acid residues, has been established as a trophic factor for the epithelial cell homeostasis (17, 18) and nutrient transport in the small intestine (19–23). Previous studies have reported that EGF inhibited the expression of NaPi-IIb (24–27), which implied that EGF inhibited the active absorption of Pi. However, EGF is known to induce repair of oxidative damage of pig small intestinal epithelial cells stimulated by lipopolysaccharide (LPS) (18). In theory, the process of the intestinal barrier repair is accompanied by increased DNA and RNA syntheses, which leads to increased phosphorus absorption in intestinal epithelial cells since phosphorus is the main element in nucleic acid synthesis. Our previous study has confirmed that EGF can promote the expression of NaPi-IIb in LPS-induced injured porcine intestinal epithelial cells (IPEC-J2) and LPS-induced injured intestine of piglets (27). It indicated that EGF could promote the active absorption of Pi under stress condition. In this review, we mainly reviewed the effect of EGF on Pi absorption and its possible mechanism, to provide a theoretical basis for the application of EGF in animal production.

## The SLC34 Family

**Figure 1:**
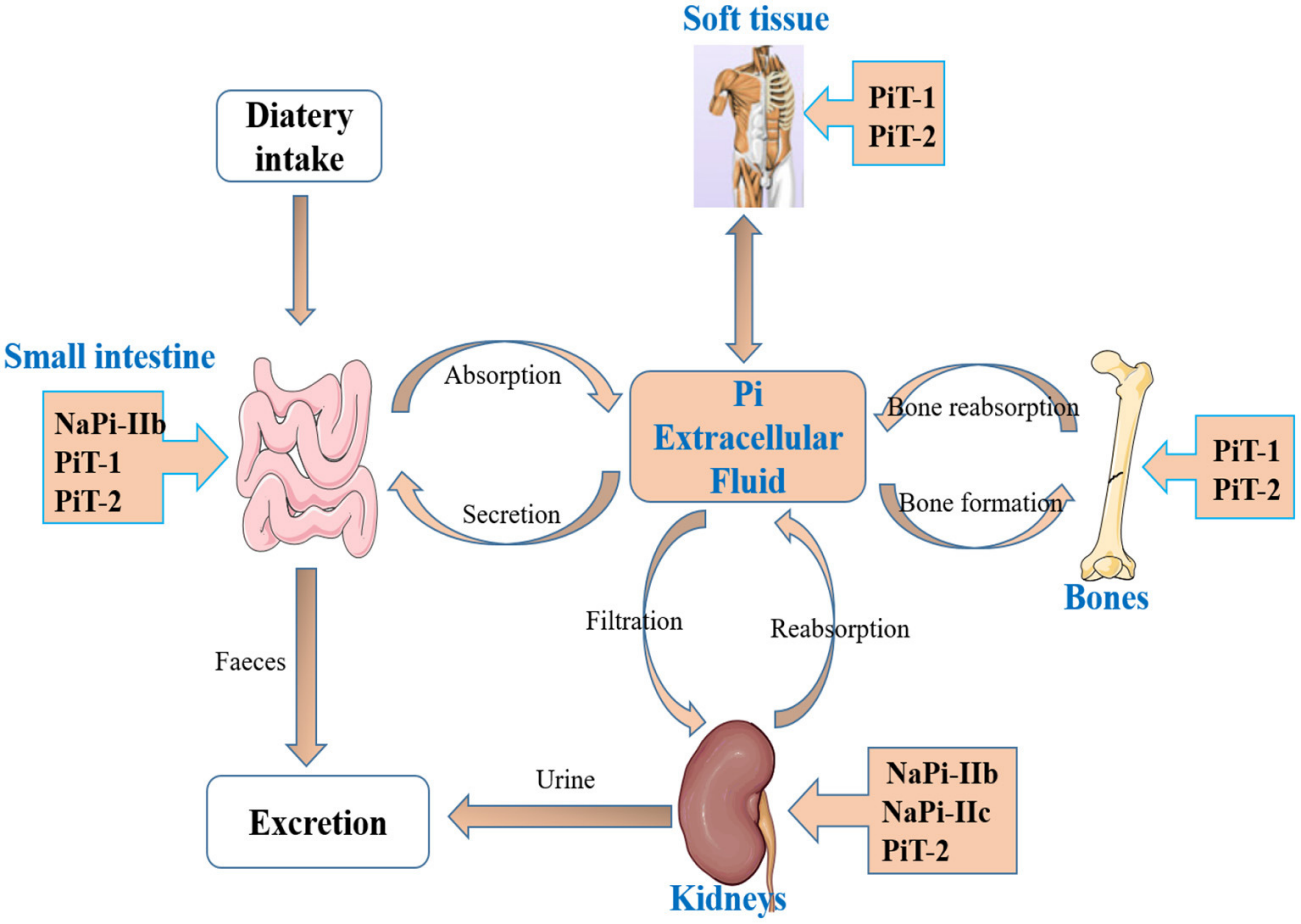
The main organs and transporters involved in inorganic phosphate (Pi) homeostasis. Pi homeostasis is regulated by the coordinated interplay of intestine, kidneys, and bones, and two families of sodium-coupled cotransporters, the SLC20 family (PiT-1, PiT-2) and the SLC34 family (NaPi-IIa, NaPi-IIb, and NaPi-IIc), involved in Pi absorption.

Pi homeostasis is regulated by the coordinated interplay of the intestine, kidneys, and bones (28, 29). The intestine absorbs Pi from the diet, kidneys reabsorb Pi from the primary urine filtrate, and the bones serve as a Pi pool, where it can be deposited as hydroxyapatite or released in case Pi supply is low (30). There are two genetically distinct families of sodium-coupled co-transporters that mediate transport of Pi in mammals, namely, the SLC20 family comprises SLC20A1 (PiT-1) and SLC20A2 (PiT-2), and the SLC34 family comprises SLC34A1 (NaPi-IIa), SLC34A2 (NaPi-IIb), and SLC34A3 (NaPi-IIc) (3, 12, 31, 32) (Figure 1). PiT-1 is widely expressed in soft tissue, small intestine, and bone. PiT-2 is widely expressed in soft tissue, small intestine, bone, and kidney. NaPi-IIa and NaPi-IIc are mainly expressed in the kidney, and NaPi-IIb is mainly expressed in the small intestine (32). However, only the physiological roles of SLC34 proteins have been extensively investigated and characterized.

SLC34 family comprises three subtypes of phosphate transporters (Table 1). NaPi-IIa is encoded by the *SLC34A1* gene, mainly expressed in the BBM of renal proximal tubular epithelial cells and is regulated by dietary Pi level, parathyroid hormone (PTH) and fibroblast growth factor (FGF23) (31–34). NaPi-IIc is encoded by the *SLC34A3* gene, which is expressed exclusively in the kidney (32, 34). The expression of NaPi-IIc is related to age, with the highest level at weaning stage, and then gradually decreases with age (35). The expression of NaPi-IIc is regulated by dietary Pi level, PTH, and FGF23 too (31–34). Previous studies have shown that NaPi-IIa and NaPi-IIc are responsible for the renal reabsorption of Pi (31–35). Beck et al. (36) found that the NaPi-IIa gene knockout (NaPi-IIa^−/−^) mouse would lead to a reduced sodium-dependent phosphorus reabsorption by about 70% in the kidney. NaPi-IIa^−/−^ mice lead to increased NaPi-IIc expression, which mediates about 30% phosphorus uptake (35, 37). However, the mechanism of renal Pi reabsorption regulated by NaPi-IIa and NaPi-IIc is different. NaPi-IIa is electrically charged and has a Na^+^:Pi ratio of 3:1, while NaPi-IIc is electrically neutral and has a Na^+^:Pi ratio of 3:1 (3, 31–35).

**Table 1:** The characteristics of SLC34 protein family.

**Gene**	**Protein**	**Substrates**	**Na^**+**^: Pi stoichiometry**	**Electrically charged**	**Main tissue distribution**
SLC34A1	NaPi-IIa	HPO_4_^2-^	3:1	+	Kidney
SLC34A2	NaPi-IIb	HPO_4_^2-^	3:1	+	Small intestine
SLC34A3	NaPi-IIc	HPO_4_^2-^	2:1	−	Kidney

“*+” means electrically charged, “−” means electrically neutral*.

NaPi-IIb is encoded by the *SLC34A2* gene, which widely expressed in lung, testicles, mammary glands, liver, salivary glands, thyroid, and small intestine, and the small intestine is the major expression site (3, 5). Like to NaPi-IIa, NAPI-IIb is also electrically charged and has a Na^+^:Pi ratio of 3:1 (3, 31–35). NaPi-IIb protein is thought to be the major sodium-dependent Pi transporter protein, since its ablation in mice abolishes Na^+^-dependent uptake of Pi (38, 39). NaPi-IIb accounts for 90% of transcellular sodium-dependent transport (38, 40, 41), which plays an important role in the intracellular Pi accumulation and Pi homeostasis. The NaPi-IIb expression *in vivo* is regulated by many physiological factors, including dietary Pi level (1, 42), calcitonin (43), 1.25(OH)_2_VD_3_ (44, 45), corticosterone (46), estrogen (47), B-RAF (48), EGF (24–27), and so on. Inhibition of intestinal NaPi-IIb expression would lead to an increased fecal phosphorus excretion, resulting in a waste of resources (30). Thus, investigating the regulatory factors of NaPi-IIb deeply is critically important for improving intestinal phosphorus utilization, decreasing manure phosphorus excretion, and reducing environmental pollution (43).

## EGF and PI Absorption

### Biological Function of EGF

Dr. Stanley Cohen first discovered EGF more than half a century ago (49). It is a small mitogenic polypeptide comprising 53 amino acid residues and three intramolecular disulfide bridges and widely exists in saliva, milk, amniotic fluid, urine, plasma, and intestinal fluid (17, 50). EGF is heat and acid stable and resistant to proteases digestion due to its special chemical structure (51), which allows its delivery to the gastrointestinal tract to exert trophic effects and makes it possible to be used in animal feed. The biological functions of EGF are mediated through binding to its receptor, EGF receptor (EGFR), a transmembrane glycoprotein, abundantly located on the apical and basolateral aspect of villus enterocytes (17, 52, 53). EGFR belongs to the transmembrane receptor tyrosine kinase of the ErbB family, with a molecular weight of 170 kDa consisting of a single polypeptide chain (54). The binding of EGF at the enterocytes surface induces dimerization of EGFR, which results in the activation of receptor tyrosine kinase (RTK) and RTK auto-phosphorylation, and subsequent activation of various signal transduction pathways, including mitogen-activated protein kinase (MAPK) (55), phosphoinositol 3 kinase (PI3K) (56), nuclear factor erythroid 2-related factor 2/ Kelch-like ECH-associated protein 1 (Nrf2/Keap1) (18), and mammalian target of rapamycin protein (mTOR) (57). Previous studies have demonstrated that EGF has many biological functions, including promoting intestinal repair (18) and nutrient absorption (23, 58, 59).

### EGF Inhibits Active Transport of Pi Under Normal Conditions

Phosphorus is an essential element for the growth and development of animals. An important physiological regulator of Pi absorption is EGF, which acts through modulation of NaPi cotransporter activity (24–27, 60, 61). Early studies in rats (60) and opossum kidney cells (61) showed that EGF inhibited renal Pi uptake by modulating NaPi-IIa cotransporter protein and mRNA levels. In intestine, previous studies have confirmed that EGF also was an important physiological regulator of Pi absorption (24–27). The study in rat and human CACO2 cells from Xu et al. (24) showed that EGF significantly inhibited the expression of *NaPi-IIb* gene. Consistent with Xu et al. (24), our previous study also found that EGF downregulated NaPi-IIb expression in IPEC-J2 cells (26), indicating the loss of active transcellular transport of Pi in the small intestine. This suggested that, under normal conditions, EGF inhibited the active transport of Pi. However, the inhibition of NaPi-IIb expression would not affect the Pi homeostasis, because the intestinal Pi absorption is the consequence of transcellular transport plus paracellular absorption (62, 63). Passive absorption through the paracellular pathway may contribute to being an alternative transport pathway to supply enough Pi for the body when a sufficient gradient of Pi is established across the epithelium (30, 63). Additionally, the compensatory mechanism of increased renal reabsorption can also result in a normal plasma Pi (30). The phenomenon of EGF promotes cell proliferation (18, 64–67) and to some extent can also demonstrate that EGF can promote phosphorus uptake. This because, in theory, during cell proliferation, more phosphorus is needed to meet the demand of DNA and RNA syntheses, but through a paracellular pathway or activation of renal compensatory mechanisms, rather than through the active transcellular transport of Pi mediated by NaPi-IIb.

### The Mechanism of EGF on NaPi-IIb Expression Regulation

EGF, as a growth hormone, plays an important role in modulating intestinal Pi absorption. Xu et al. (24) reported that EGF affected *NaPi-IIb* gene expression by inhibiting transcriptional activation in CACO2 cells. Further study indicated that EGF downregulated *NaPi-IIb* gene expression is through regulating the binding of transcription factor c-myb and *NaPi-IIb* gene promoter. The EGF response region was located in the promoter between −784 and −729 base pair (bp) of the promoter of human, and the downregulation of promoter function is mediated by EGF-activated protein kinase C/protein kinase A PKC/PKA and MAPK pathways (25). Previous work in our laboratory showed that the EGF response region was located in the −1,092 to −1,085 bp region (5′-TCCAGTTG-3′) in porcine intestinal epithelial cells, IPEC-J2 (26). Further studies showed that EGF downregulated the expression of NaPi-IIb in IPEC-J2 cells by activating signaling molecules such as EGFR, PKA, PKC, P38, extracellular regulated protein kinases (ERK), and c-Jun N-terminal kinase (JNK) (68). Although previous studies had proved that EGF-activated MAPK, PKC, and PKA pathways are all involved in the regulation of NaPi-IIb in intestinal epithelial cells (25, 68), how their downstream signaling molecules ultimately regulate the expression level of NaPi-IIb remains unknown.

### EGF Promotes Active Transport of Pi Under Intestinal Injury Condition

The intestinal tract is not only the main part of animal nutrition digestion and absorption but also acts as a physical and immunological protective barrier against foreign antigens and pathogens (17, 69–71). The integrity of intestinal is the foundation of nutrition absorption for animals (72). However, the intestinal epithelium homeostasis of animals is usually affected by bacterial infection, endotoxin challenge, weaning stress, and oxidative stress, which can lead to intestinal damage and intestinal barrier function dysfunction (73–76). EGF has been established as a trophic factor for epithelial cell homeostasis (17, 18) and nutrient transport in the small intestine (22, 58, 66, 77, 78). Previous researches have demonstrated that EGF was able to attenuate the intestinal mucosal epithelial cells injury as well as promotes the repair of damaged mucosa epithelium (18, 79–82). In theory, during the process of injured intestine repairing, more phosphorus is needed to meet the demand of DNA and RNA syntheses. Previous studies had shown that in some disease states, such as hyperphosphatemia induced by intestinal ischemia/injury, serum Pi levels and EGF levels were increased (83, 84), which indicated that EGF might play a role in regulation of Pi homeostasis in response to intestinal injury. However, it is not clear whether EGF mediates the active transport of Pi by regulating the expression of NaPi-Iib, since the regulation of Pi is a complex network, which is achieved by the combined action of intestine, kidneys, and bones (10, 85–87). Our previous study showed that EGF could promote the expression of NaPi-IIb expression in LPS-induced IPEC-J2 cells and the jejunum and ileum of LPS-induced piglets (27). It indicated that under intestinal injury condition, EGF could release the inhibition of NaPi-IIb and regulate the active absorption of Pi mediated by NaPi-IIb to meet the body's need for phosphorus and accelerate the process of intestinal repair. However, there is still a lack of researches on EGF regulation of intestinal Pi uptake under other injury conditions, like intestinal ischemia/injury, inflammatory bowel diseases, and necrotizing enterocolitis. In addition, the mechanism of EGF on NaPi-IIb-mediated Pi uptake under intestinal injury condition remains unclear, which needs to be further research.

## Conclusions

**Figure 2:**
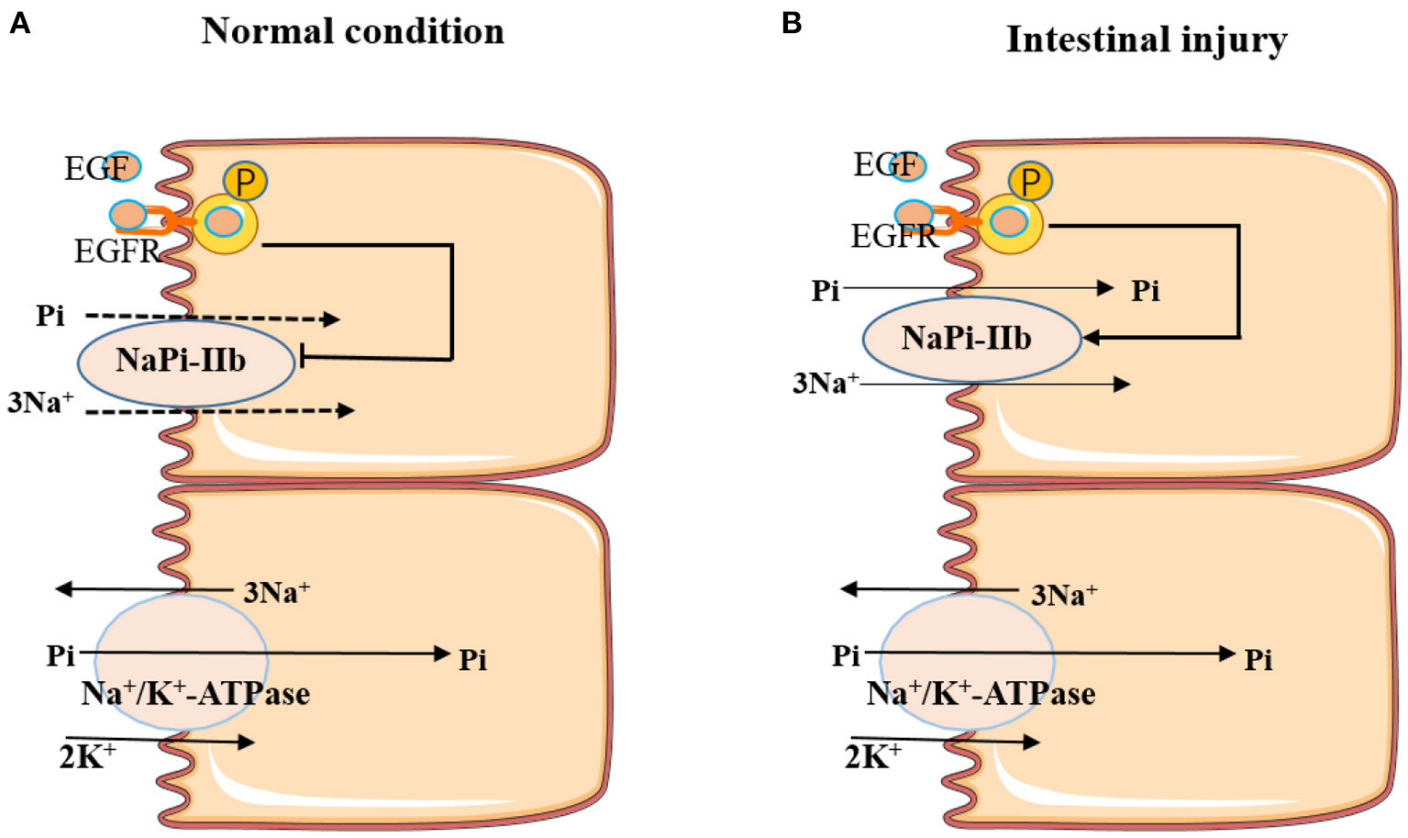
Effects of EGF on active Pi-transport-mediated NaPi-IIb under different conditions. **(A)** EGF inhibited the active transport of Pi by inhibiting the expression of NaPi-IIb under normal conditions. **(B)** EGF promoted the active absorption of Pi through upregulating the expression of NaPi-IIb under intestinal injury condition.

In summary, EGF is involved in regulating intestinal Pi absorption, and the role of EGF in modulating intestinal Pi absorption depends on the physiological status of the animal. Under normal conditions, EGF inhibited the active transport of Pi through activating MAPK, PKC, and PKA pathways to inhibit the expression of NaPi-IIb. While, under intestinal injury condition, EGF could promote the active absorption of Pi through upregulating the expression of NaPi-IIb (Figure 2). Further studies could focus on how EGF regulates the expression of NaPi-IIb under intestinal injury condition, thereby promoting the active transport of intestinal Pi.

## Author Contributions

XT: conceptualization and writing—original draft preparation. XT and XL: methodology and supervision. XT, HL, and XL: formal analysis and data curation. XT and HL: resources, writing—review and editing, and project administration. All authors have read and agreed to the published version of the manuscript.

## Conflict of Interest

The authors declare that the research was conducted in the absence of any commercial or financial relationships that could be construed as a potential conflict of interest.
